# Temporal dynamics of nutrient elements in biochar and biochar-amended soils over three years: a comparative micro-XRF and SEM–EDX study

**DOI:** 10.1038/s41598-026-59314-z

**Published:** 2026-06-21

**Authors:** Suphathida Aumtong, Phruetthiphong Soongsoongnoen, Dechatorn Wanwinit

**Affiliations:** Department of Soil Science, Faculty of Agricultural Production, Maejo University, Chiang Mai, 50290 Thailand

**Keywords:** Biochar aging, Micro-XRF, SEM–EDX, Fe–Al redistribution, Organo-mineral coatings, Effective microorganisms, Biogeochemistry, Ecology, Ecology, Environmental sciences

## Abstract

Biochar reshapes soil composition for years, yet most evidence comes from short-term, single-element laboratory incubations, leaving multi-element dynamics in tropical soils poorly resolved. We tracked 11 elements in longan-wood biochar and biochar-amended Ultisols over three years after a single field application, combining micro-XRF with SEM–EDX (one Map Sum Spectrum per group). Soil Fe and Al showed a non-linear response: both peaked at Year 1 (Fe 17.77 ± 3.93; Al 21.33 ± 1.88 wt.%; ~ 3.1- and 1.75-fold above control), fell at Year 2, then rose again at Year 3—a pattern invisible to single time-point studies, consistent with organo-mineral coating formation and reworking. Soil P stayed below detection despite annual fertilisation, indicating persistent Fe–Al phosphate fixation, while soil K peaked at Year 2 (1.82 wt.%). The biochars were Ca-rich (> 60 wt.%), acting as strong liming agents. EM-inoculated biochar showed higher surface carbon (92.7 vs. 77.6 wt.%), consistent with microbial biofilm deposition, though EM did not alter inorganic composition; a high Year-3 N signal (13.52 ± 23.42 wt.%) is a semi-quantitative artefact, not a reliable soil-N value. These findings reveal non-linear multi-element redistribution from a single biochar application and show the value of pairing bulk and surface analysis for long-term biochar–soil studies.

**Supplementary Information:**

The online version contains supplementary material available at 10.1038/s41598-026-59314-z.

## Introduction

Biochar—the carbonaceous solid produced by pyrolysis of organic biomass under limited oxygen—has attracted considerable interest as a soil amendment for carbon sequestration, soil improvement, and nutrient availability enhancement^1–3^. Once incorporated into soil, it undergoes progressive physical, chemical, and biological aging over months to decades, altering surface chemistry, mineral associations, and nutrient release characteristics^2,4–6^.

Pyrolysis concentrates inorganic macronutrients—particularly Ca, K, P, and Mg—in the solid residue as volatile C and N are lost, generating ash-rich materials with strong liming potential^3,7,8^. Biochar is increasingly co-applied with microbial inoculants, particularly effective microorganism (EM) consortia, to exploit synergistic effects on soil biological activity and nutrient cycling^1,9,10^. Whether such inoculation alters the inorganic elemental composition of biochar remains an open question.

Previous studies on biochar-amended soils have largely focused on single or paired elements (commonly N and P) over short incubation periods, or relied on a single method such as chemical digestion or ^15^N isotope tracing^3,11,12^. While valuable for quantifying plant-available nutrient pools, these approaches offer limited insight into concurrent multi-element redistribution across the soil–biochar interface over multi-year timescales. The mechanistic linkage between biochar mineralogy and long-term soil elemental dynamics therefore remains poorly resolved.

Despite growing interest in biochar aging, multi-year field studies that resolve both bulk and surface-level elemental changes simultaneously remain rare. Most published studies rely on laboratory incubations (< 1 year) or single-method characterisation. Few have been conducted in tropical Ultisols, which present distinct geochemical challenges: high Al and Fe activity, low pH buffering capacity, and strong P fixation^3^. Although a combined micro-spectroscopic and electron-microscopic approach has been advocated for comprehensive biochar characterisation^14,15^, its application to resolve bulk and near-surface elemental composition has rarely been deployed in long-term field datasets from tropical systems.

The present study addresses these gaps by applying micro-X-ray fluorescence (micro-XRF) and scanning electron microscopy with energy-dispersive X-ray spectroscopy (SEM–EDX) to characterise elemental composition in biochar and biochar-amended soils over three years. This dual-method approach captures both area-averaged elemental redistribution (micro-XRF, n = 3 field replicates per soil application age) and near-surface elemental composition (SEM–EDX, single representative Map Sum Spectra per group) across the biochar–soil interface. Such multi-element, multi-year spectroscopic datasets are rare, and the replicated field design for soils (n = 3 trees per application age) represents a methodological advance over single-method studies^14,15^.

The two methods address different analytical scales and thus capture complementary aspects of biochar elemental dynamics. Micro-XRF maps elemental signals over a millimetre-scale area of the intact, un-pelletised specimen presented directly to the beam in air, making it suitable for detecting broad compositional shifts such as Fe–Al redistribution or Si-mineral dilution at that scale; because it is acquired in air, light-element signals (notably N) are strongly attenuated and only semi-quantitative. SEM–EDX, by contrast, interrogates elemental composition within a shallow interaction volume (~ 1–2 µm at 15 kV), making it sensitive to surface-bound mineral phases, organic coatings, and microbial deposits. SEM–EDX additionally registers C and O—the dominant elements in biochar—which are not quantified by micro-XRF in this configuration. Together, the two methods span complementary scales: micro-XRF provides area-averaged inorganic composition, while SEM–EDX captures near-surface elemental composition at the biochar–soil interface. Deploying these two complementary methods across spatial scales in a multi-year field dataset is therefore a further aim of the present study.

## Results

### Macronutrient composition of biochar treatments

Both biochar treatments were markedly enriched in plant macronutrients relative to all soil groups (Supplementary Fig. S1; Table 1). Full elemental composition for all 11 elements is provided in Supplementary Table S1. Mean Ca concentrations exceeded 60 wt.% (Biochar + EM: 62.16 ± 6.19 wt.%; Biochar: 60.39 ± 4.61 wt.%), substantially higher than any soil group (< 0.85 wt.%). K was similarly elevated (Biochar + EM: 11.76 ± 6.93 wt.%; Biochar: 15.85 ± 4.27 wt.%), as were P (≥ 5.29 wt.%) and Mg (~ 6.5 wt.%). All soil treatments contained Ca < 0.90 wt.%, K < 1.90 wt.%, and P below detection. Differences among the soil application ages were large and statistically significant for four of these elements: Si (F = 6.47, p = 0.016), Fe (F = 6.50, p = 0.015), Al (F = 8.35, p = 0.008), and K (F = 5.49, p = 0.024); Ca, Mg, Ti and Mn did not differ significantly across the four soil application ages (all df = 3, 8; n = 3 per soil age); P, N and S were below detection or only semi-quantitatively resolved in soil and are not interpreted as quantitative concentrations. The Biochar versus Biochar + EM contrast, based on a single specimen per treatment measured in triplicate, is reported descriptively only. SEM–EDX surface analysis showed the same qualitative macronutrient-enrichment pattern in the biochars (Table 2).

**Table 1:** Mean elemental composition (wt.%) of six treatment groups determined by micro-XRF (mean ± s.d., n = 3 per group). Mean shown above ± s.d. in each cell. See footnote for element symbols, detection convention, and ANOVA results.

Treatment	Ca	K	P	Mg	Si	Fe	Al	N	Ti	Mn	S
Biochar + EM	**62.16** ± 6.19	**11.76** ± 6.93	**5.29** ± 1.80	**6.30** ± 1.01	**5.32** ± 1.77	**0.99** ± 0.66	**0.51** ± 0.12	**6.69** ± 11.58	**0.18** ± 0.11	**0.12** ± 0.01	**0.48** ± 0.24
Biochar	**60.39** ± 4.61	**15.85** ± 4.27	**5.62** ± 0.37	**6.66** ± 0.36	**5.72** ± 0.80	**0.88** ± 0.42	**0.55** ± 0.07	**3.45** ± 5.98	**0.20** ± 0.04	**0.10** ± 0.01	**0.31** ± 0.03
Soil 0 yr	**0.61** ± 0.57	**1.48** ± 0.07	**0.00** ± 0.00	**0.15** ± 0.03	**77.40** ± 0.70	**5.68** ± 1.02	**12.19** ± 0.52	**0.00** ± 0.00	**2.04** ± 0.11	**0.25** ± 0.05	**0.00** ± 0.00
Soil 1 yr	**0.85** ± 0.06	**1.57** ± 0.02	**0.00** ± 0.00	**0.16** ± 0.01	**55.77** ± 5.39	**17.77** ± 3.93	**21.33** ± 1.88	**0.00** ± 0.00	**1.95** ± 0.29	**0.20** ± 0.17	**0.00** ± 0.00
Soil 2 yr	**0.63** ± 0.17	**1.82** ± 0.07	**0.00** ± 0.00	**0.12** ± 0.10	**73.12** ± 7.31	**8.30** ± 4.09	**13.34** ± 3.22	**0.00** ± 0.00	**1.99** ± 0.15	**0.30** ± 0.07	**0.00** ± 0.00
Soil 3 yr	**0.56** ± 0.48	**1.49** ± 0.21	**0.00** ± 0.00	**0.14** ± 0.05	**50.72** ± 15.18	**13.74** ± 4.60	**18.09** ± 3.43	**13.52** ± 23.42	**1.38** ± 0.44	**0.10** ± 0.12	**0.01** ± 0.01

Bold values denote the reported mean concentrations and carry no statistical significance.

† Element symbols: Ca, K, P, Mg, Si, Fe, Al, N, Ti, Mn, S correspond to calcium, potassium, phosphorus, magnesium, silicon, iron, aluminium, nitrogen, titanium, manganese, sulfur respectively. All values in normalised wt.%; below detection shown as 0.00. Soil-only four-group temporal ANOVA (df = 3, 8): Si p = 0.016 (η^2^ = 0.71); Fe p = 0.015 (η^2^ = 0.71); Al p = 0.008 (η^2^ = 0.76); K p = 0.024 (η^2^ = 0.67); Ca, Mg, Ti, Mn not significant; P, N, S not robustly quantified in soil. Biochar vs. Biochar + EM is descriptive only (single specimen per biochar, technical triplicates).

**Table 2:** Near-surface elemental composition (wt.%) of six treatment groups determined by SEM–EDX (single Map Sum Spectrum per group, n = 1; carbon- and oxygen-free renormalised basis). Surface C values for biochars are reported separately below the table; instrumental and acquisition details are given in Methods 4.4.

Treatment	Ca	K	P	Mg	Si	Fe	Al	N	Cu
Biochar + EM	**43.84**	**12.33**	**15.07**	**15.07**	**12.33**	**n.d**	**n.d**	**n.d**	**n.d**
Biochar	**33.33**	**20.29**	**18.84**	**14.49**	**11.59**	**n.d**	**1.45**	**n.d**	**n.d**
Soil 0 yr	**0.30**	**0.70**	**0.00**	**0.70**	**34.10**	**2.90**	**17.60**	**41.30**	**1.30**
Soil 1 yr	**0.49**	**n.d**	**0.00**	**1.22**	**51.34**	**6.11**	**41.32**	**0.00**	**0.00**
Soil 2 yr	**0.60**	**n.d**	**0.00**	**2.30**	**86.50**	**7.60**	**n.d**	**0.00**	**3.00**
Soil 3 yr	**1.10**	**n.d**	**0.00**	**3.40**	**78.10**	**17.00**	**n.d**	**0.00**	**0.40**

Bold values denote the reported mean concentrations and carry no statistical significance.

Surface organic carbon (C-inclusive basis, biochars only): Biochar+EM = 92.7 wt.% C; Biochar = 77.6 wt.% C (with O = 15.5 wt.%). These C-inclusive values are reported separately from the C/O-free renormalised data above as direct evidence of microbial biofilm deposition on Biochar+EM surfaces; against the bulk-biochar TOC of 29.73 wt.% (IQS dry-combustion; Supplementary Table S5), these correspond to surface-to-bulk carbon ratios of ~3.1× and ~2.6× respectively; because EDX carbon is semi-quantitative on uncoated biochar, these ratios are indicative rather than absolute.

† n.d., element not detected in the spectrum; 0.00, quantified but below numerical precision. Within-group dispersion is not estimable from single spectra (see Table 1 for replicated micro-XRF data, n = 3). Minor elements detected by SEM–EDX but not shown in the column set: Ti and Mn in Soil 0 yr only (1.1 and 0.2 wt.% respectively); S in Biochar + EM only (1.37 wt.%); below detection in all other groups.

The SEM–EDX dataset also shows that macronutrient concentrations on biochar surfaces (Ca, K, Mg) were elevated in both biochar Map Sum Spectra (Table 2), consistent with the persistence of inorganic mineral phases on biochar particle surfaces over the study period. This surface-level enrichment is consistent with the persistence of thermally concentrated inorganic phases on biochar surfaces and may reflect their mobilisation from interior pore surfaces towards the particle exterior through dissolution and diffusion; because each biochar was characterised by a single Map Sum Spectrum, this is offered as a hypothesis rather than a demonstrated temporal sequence^17,18^.

### Effect of EM inoculation

Because each biochar treatment was represented by a single specimen measured in triplicate (technical replicates), the Biochar and Biochar + EM compositions are compared descriptively rather than tested statistically. The two biochars were closely similar across all micro-XRF-measured elements, with no consistent difference attributable to EM inoculation, indicating that EM inoculation did not measurably alter the inorganic elemental composition. SEM–EDX analysis indicated higher surface-associated C in Biochar + EM (92.7 wt.%) relative to Biochar (77.6 wt.%), consistent with the accumulation of microbial biomass and extracellular polymeric substances on biochar surfaces during the 14-day incubation^1,19^.

### Temporal dynamics of soil elemental composition

**Fig. 1:**
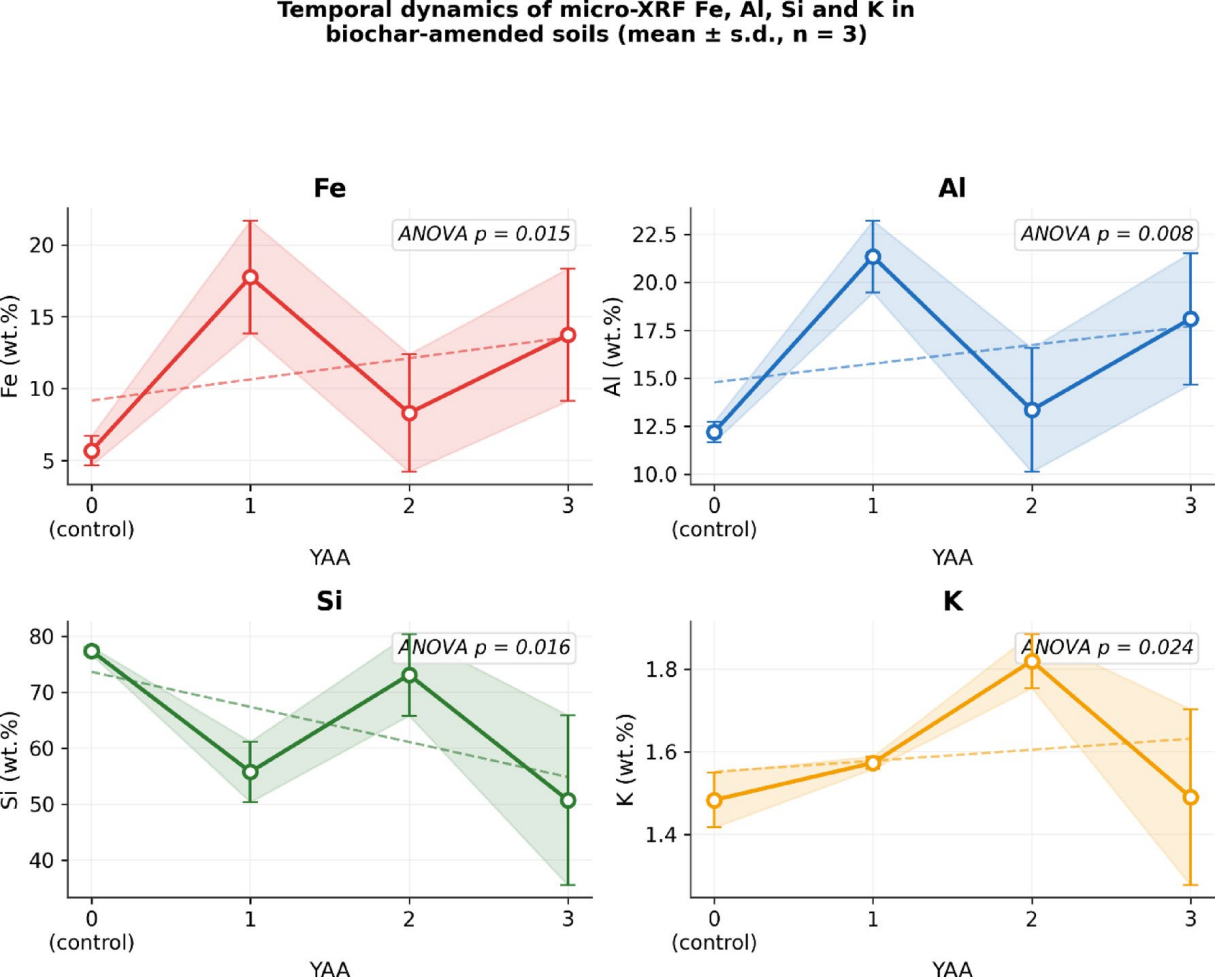
Temporal dynamics of micro-XRF-determined Fe, Al, Si, and K concentrations (wt.%) in biochar-amended soils at 0, 1, 2, and 3 years after a single biochar application (15 kg tree⁻^1^ (≈ 2.34 t ha⁻^1^)). Lines connect group means; shaded bands =  ± s.d. (n = 3); dashed lines = linear trend. Soil-only one-way ANOVA (df = 3,  8) p-values shown in each panel. YAA = years after application.

Temporal patterns of soil elemental composition over 0–3 years after biochar application are presented in Table 3 and Fig. 1. Fe rose from 5.68 ± 1.02 wt.% (Soil 0 yr) to 17.77 ± 3.93 wt.% at Year 1—a ~ 3.1-fold increase—declining to 8.30 ± 4.09 wt.% at Year 2 before partially recovering to 13.74 ± 4.60 wt.% at Year 3. Al followed a parallel trajectory, peaking at 21.33 ± 1.88 wt.% at Year 1 (vs. 12.19 ± 0.52 wt.% in controls), declining at Year 2 (13.34 ± 3.22 wt.%), and rising again at Year 3 (18.09 ± 3.43 wt.%).

**Table 3:** Temporal dynamics of micro-XRF elemental concentrations (mean ± s.d., wt.%, n = 3) in biochar-amended soils at 0, 1, 2, and 3 years after a single biochar application (15 kg tree⁻^1^ (≈ 2.34 t ha⁻^1^)).

Time point	Si (wt.%)	Fe (wt.%)	Al (wt.%)	Ca (wt.%)	K (wt.%)	Mg (wt.%)	Ti (wt.%)	Mn (wt.%)
Soil 0 yr	**77.40** ± 0.70	**5.68** ± 1.02	**12.19** ± 0.52	**0.61** ± 0.57	**1.48** ± 0.07	**0.15** ± 0.03	**2.04** ± 0.11	**0.25** ± 0.05
Soil 1 yr	**55.77** ± 5.39	**17.77** ± 3.93	**21.33** ± 1.88	**0.85** ± 0.06	**1.57** ± 0.02	**0.16** ± 0.01	**1.95** ± 0.29	**0.20** ± 0.17
Soil 2 yr	**73.12** ± 7.31	**8.30** ± 4.09	**13.34** ± 3.22	**0.63** ± 0.17	**1.82** ± 0.07	**0.12** ± 0.10	**1.99** ± 0.15	**0.30** ± 0.07
Soil 3 yr	**50.72** ± 15.18	**13.74** ± 4.60	**18.09** ± 3.43	**0.56** ± 0.48	**1.49** ± 0.21	**0.14** ± 0.05	**1.38** ± 0.44	**0.10** ± 0.12

Bold values denote the reported mean concentrations and carry no statistical significance.

† All values in normalised wt.%. YAA = years after application.

The Year-1 Al maximum resolved by micro-XRF was qualitatively corroborated by the SEM–EDX surface analysis (Al = 41.32 wt.% at Year 1; Table 2). The SEM–EDX Fe pattern was less aligned with the micro-XRF Year-1 Fe peak (SEM–EDX Fe rose monotonically through Year 3), reflecting the limits of single-spectrum sampling (n = 1) for resolving temporal trends in spatially heterogeneous Fe phases rather than necessarily indicating divergent geochemistry. This pattern is consistent with a conceptual model of progressive organo-mineral interaction, whereby Fe–Al phases may first accumulate on reactive biochar surfaces (Year 1 peak), then partially dissolve and reprecipitate as more crystalline phases in the bulk soil matrix (Year 2 decline), before re-engaging with biochar surfaces as fresh pore networks are exposed through physical fragmentation of aging biochar particles (Year 3 secondary increase)^5,6,17,20^. Analogous Fe–Al accumulation and structural transformation at aged biochar–mineral interfaces has been documented by microscopy and spectroscopic studies^21,22^.

The high apparent N concentration in Soil 3 yr by micro-XRF (13.52 ± 23.42 normalised wt.%, n = 3; one replicate reaching 40.56 wt.% while the other two returned N = 0) warrants specific comment. This elevated, highly variable signal is presented as a limitation rather than a reliable measurement. Independent IQS Kjeldahl analysis of the biochar batch (Supplementary Table S5) gave a bulk nitrogen content of 0.79 wt.%, whereas the micro-XRF biochar N values (BC + EM: 6.69 ± 11.58 wt.%; BC: 3.45 ± 5.98 wt.%) overestimate the true content by roughly an order of magnitude; likewise, the SEM–EDX soil N of 41.30 wt.% in Soil 0 yr (Table 2) is a normalisation artefact of the C/O-free basis^26^. These light-element signals should be read as semi-quantitative artefacts rather than absolute concentrations^26,27^, and reliable soil-N quantification requires independent combustion- or digestion-based methods (Kjeldahl or dry-combustion carbon–hydrogen–nitrogen analysis). The large within-group variance (s.d. = 23.42 wt.%)—driven by a single replicate returning an implausibly high value (40.56 wt.%, far above any realistic soil-N content) while the other two returned zero—is characteristic of an unreliable, semi-quantitative signal rather than a robust measurement. Genuine micro-scale N heterogeneity (for example, localised fertiliser-N retained in organic-rich microsites) cannot be excluded, but it cannot be distinguished from measurement artefact with the present data^23,24,28^.

Si decreased from 77.40 ± 0.70 wt.% in Soil 0 yr to 55.77 ± 5.39 wt.% at Year 1, partially recovering at Year 2 (73.12 ± 7.31 wt.%) before declining further to 50.72 ± 15.18 wt.% at Year 3. Ca, K, and Mg in soil showed only modest variation across all time points, while P and S remained below the micro-XRF detection limit in all soil groups. Ti and Mn varied modestly across the chronosequence (Ti: 1.38–2.04 wt.%; Mn: 0.10–0.30 wt.%) without statistically significant temporal trends.

Soil K concentrations showed a modest but consistent temporal pattern throughout the study period, rising from 1.48 ± 0.07 wt.% (Soil 0 yr) to a peak of 1.82 ± 0.07 wt.% at Year 2, before declining to 1.49 ± 0.21 wt.% at Year 3. This temporal pattern broadly coincides with inter-annual variations in K₂O fertiliser inputs: Year 0 received the highest K₂O input (81 g tree⁻^1^ yr⁻^1^ from the 15–15-15 and 13–13-21 blend), Year 2 the lowest (30 g tree⁻^1^ yr⁻^1^ under the high-N urea-based regime), and Year 3 an intermediate rate (41 g tree⁻^1^ yr⁻^1^). The progressive increase in soil K through Year 2 likely reflects the cumulative contribution of both biochar-derived K release and annual K fertiliser inputs, as biochar has been shown to increase total soil K via slow release of ash-associated K phases and suppression of K leaching^29,30^. The partial decline at Year 3 likely reflects reduced fertiliser K inputs and increased plant uptake^31^. The narrow absolute range (1.48–1.82 wt.%) relative to the higher K enrichment in biochar (11.76–15.85 wt.%) confirms that biochar-derived K constitutes a minor fraction of total soil K at this application rate^31^. As micro-XRF measures total K, the exchangeable (plant-available) K pool—typically only a small percentage of total K—is not resolved here and would require selective extraction, as noted above for P.

**Fig. 2:**
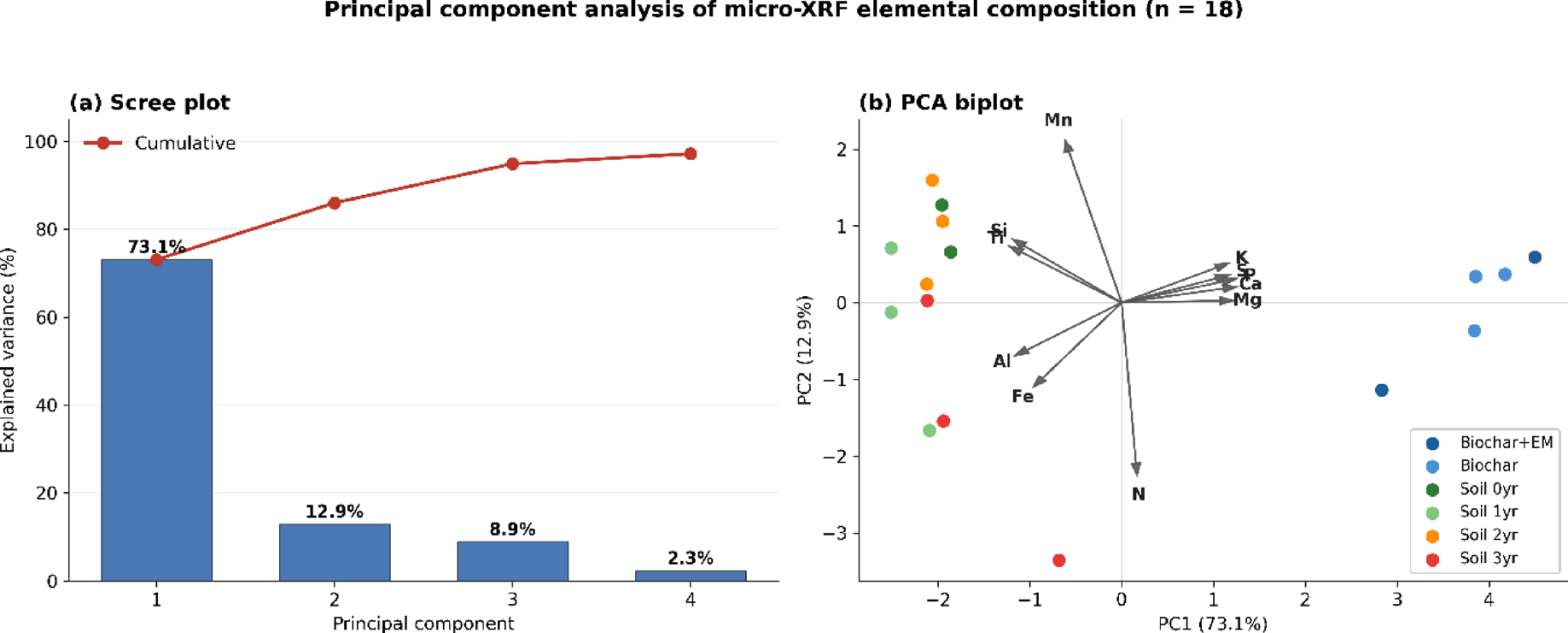
Principal component analysis of micro-XRF elemental composition data (n = 18). **(a)** Scree plot showing variance explained per PC and cumulative variance (red line). **(b)** PCA biplot of PC1 vs. PC2 scores with element loading vectors. Vectors shown only for elements with |loading|≥ 0.25 on PC1 or PC2. BC + EM = Biochar + EM; BC = Biochar; S-0 yr to S-3 yr = soil 0–3 years after application. PC1 + PC2 explain 86.0% of the total variance (PC1 73.1%, PC2 12.9%).

Principal component analysis of the micro-XRF dataset (Fig. 2) condensed the 11-element composition onto two components that together captured 86.0% of the total variance (PC1: 73.1%; PC2: 12.9%). The biplot showed clear separation of the two biochar treatments from the four soil groups along PC1, reflecting the contrast between biochar (high Ca, K, P, Mg) and mineral soil (high Si, Fe, Al); additional separation among the soil application ages was captured along PC2, consistent with the transient Year-1 Fe–Al enrichment described above.

## Discussion

The exceptionally high Ca concentrations in both biochar treatments (> 60 wt.%) reflect thermodynamic concentration of Ca-rich inorganic phases during pyrolysis and are characteristic of Ca-rich feedstocks^3,7,11^. Such biochars act as potent liming agents, raising soil pH, cation exchange capacity (CEC), and base cation availability when applied to acidic soils^11,32^. The near-zero P in all soil treatments despite ∼5 wt.% P in biochar reflects dilution at the 15 kg tree⁻^1^ (≈ 2.34 t ha⁻^1^) application rate; companion soil-extraction data (Supplementary Table S6) showed that extractable plant-available P in the topsoil was higher by Year 3 (mean 210 to 466 mg kg⁻^1^, an approximately 2.2-fold increase), although high spatial variability meant the difference was not statistically significant (one-way ANOVA, p = 0.16); this suggests, but does not confirm, gradual P-availability gains consistent with slow release of biochar-associated Ca-P and Mg-P phases^12,33^.

The persistent absence of detectable P in all soil groups across all four time points is striking given that P₂O₅ fertiliser was applied annually at 30–63 g tree⁻^1^. This is consistent with P behaviour in acidic Ultisols, where added or biochar-released phosphate is rapidly immobilised onto Fe and Al (oxy)hydroxide surfaces as sparingly soluble Fe–P and Al–P complexes. As micro-XRF reports total P, the persistent below-detection signal reflects a low total soil-P concentration (modest inputs diluted within an inherently low-P Ultisol); fixation, by contrast, governs the plant-available P pool, which micro-XRF cannot resolve and which requires selective extraction^16,34–36^. This has two implications. First, micro-XRF bulk analysis is not suitable for tracking plant-available P in Fe/Al-rich tropical Ultisols; Olsen or Bray extraction or P fractionation are required. Second, it indirectly supports biochar’s role in ameliorating P fixation: as biochar raises pH and supplies Ca and Mg as competing cations, it may gradually shift P from recalcitrant Fe–P and Al–P towards more labile Ca–P and Mg–P fractions^12,37^, even when unresolvable by bulk XRF. The transiently elevated Fe and Al at Year 1 therefore have agronomic significance beyond mineralogy: they may represent a temporary period of maximal P-fixation capacity, consistent with progressive organo-mineral transformation. Because Fe and Al rose again at Year 3, fixation capacity is itself non-monotonic; any improvement in plant-available P over time would therefore more likely reflect biochar-driven P release and pH/cation effects—and the lower P-reactivity of increasingly crystalline Fe–Al phases—rather than a simple decline in total Fe–Al^16,35^.

The Ca concentrations observed here (> 60 wt.%) are markedly higher than values typically reported for wood-derived biochars (generally < 10 wt.%) and are more consistent with Ca-rich feedstocks such as bone char or Ca-enriched manure-derived materials^11^. This is agronomically important: the liming effect of such Ca-rich biochar is expected to persist for several years before gradual re-acidification as alkaline minerals dissolve and base cations are leached or taken up^32^. The present three-year dataset captures the early-to-mid phase of this liming trajectory^5,6^. Three factors contribute to the Ca-rich profile: (i) longan wood is a tropical hardwood that accumulates more Ca than the temperate softwoods used in many biochar studies^3,8^; (ii) pyrolysis at 400–500 °C volatilises C and N while retaining alkaline-earth phases, concentrating Ca in the ash-rich solid residue (bulk ash content 8.73 wt.%; Supplementary Table S5)^3,8^; and (iii) the carbon- and oxygen-free renormalisation adopted for micro-XRF (Sect. “micro-XRF elemental analysis”) further inflates the apparent Ca concentration. Because the independent IQS dry-combustion analysis gave a bulk biochar TOC of 29.73 wt.% (Supplementary Table S5), and biochar oxygen typically adds a further ~ 10–15 wt.%, this renormalisation inflates the detected inorganic fractions by ~ 1.7-fold relative to a C/O-inclusive basis; the Ca content on a C/O-inclusive basis is therefore ~ 35–40 wt.%, still elevated for wood biochar. For soils, where total organic carbon is small (1–2 wt.%), the renormalisation effect is negligible, so soil values are comparable on both bases while biochar values should be compared only with other C/O-free renormalised datasets. Consistent with this, the biochar mineral concentrations measured here by micro-XRF and SEM–EDX (Ca, K and Mg) are roughly an order of magnitude higher than the corresponding bulk values obtained by independent ICP-OES analysis (Supplementary Table S5), because the spectroscopic methods preferentially sample mineral-enriched surfaces and report semi-quantitative values on a carbon- and oxygen-free renormalised basis, whereas ICP-OES digests the carbon-dominated bulk; the two are therefore complementary surface-versus-bulk views rather than equivalent concentrations.

The SEM–EDX dataset (Table 2; side-by-side comparison in Supplementary Table S2) comprises a single Map Sum Spectrum per group (n = 1) on a carbon- and oxygen-free basis, sampling a shallow micrometre-scale volume, whereas micro-XRF provides millimetre-area-averaged values from the intact specimen. The two datasets therefore sample fundamentally different spatial scales and are compared qualitatively rather than directly. Qualitatively, both methods identify the same dominant elements–high Si with substantial Fe and Al in soils, and high Ca in biochars–and the SEM micrographs confirm that aged biochar–soil particles are markedly heterogeneous, so near-surface and area-averaged values are expected to differ in detail. As a quantitative check, Spearman rank correlation between micro-XRF means and SEM–EDX values for the eight shared elements across all six groups (n = 48; Supplementary Table S7) was moderate-to-strong (ρ = 0.585, p < 10⁻^4^; Pearson r = 0.809, p < 10⁻^11^), rising to ρ = 0.844 (p < 10⁻⁸) for pairs with a non-zero signal in both methods (n = 30). Concordance was strongest for P (ρ = 1.00) and Fe (ρ = 0.81), intermediate for Ca, K, Si and Mg (ρ ≈ 0.5–0.6), and weakest for Al (ρ = 0.33) and N (ρ =  − 0.42). These patterns reinforce the qualitative interpretation: the methods consistently distinguish the biochar-vs-soil contrast (P, Fe, Ca, K) but diverge on light-element (N) quantification and on the temporal Al pattern that single-spectrum (n = 1) SEM–EDX cannot resolve. They are complementary rather than interchangeable^14,26^.

The complementary use of micro-XRF and SEM–EDX adopted here contrasts with most published biochar-aging studies, which rely on bulk analysis or surface characterisation alone. Reliable surface microanalysis of field-aged biochar requires selecting regions free of adhering soil particles to avoid elemental contamination, together with appropriate statistical handling of multi-timepoint data. The broad major-element agreement between the two methods is consistent with validation work showing that standardless ZAF (atomic number, absorption, fluorescence) correction in SEM–EDX reproduces major-element ratios when sample heterogeneity is controlled^26^.

A note on analytical approach is warranted, as raised in review. micro-XRF measures total elemental composition non-destructively on intact specimens; because each measurement is an area-averaged map (2–5 mm field; Sect. “micro-XRF elemental analysis”) acquired on three independent field replicates per soil age, it yields a spatially representative average for heterogeneous biochar–soil materials rather than a single-point value, which is well suited to non-uniform samples. This differs in kind from wet-chemical approaches: acid digestion gives total dissolved concentrations but is destructive and may incompletely solubilise refractory aluminosilicate phases, whereas selective leaching (e.g., Olsen or Bray extraction, or exchangeable-cation extraction) reports operationally-defined plant-available fractions rather than total content. The two families are therefore complementary rather than equivalent–total and spatially-resolved (micro-XRF) versus extractable and bulk-averaged (wet-chemical). This is most consequential for P: micro-XRF reports total P, which is below detection here because the total soil-P concentration is low, whereas the agronomically critical plant-available P—governed by fixation onto Fe/Al phases—requires selective extraction to quantify. Acid-digestion or ICP cross-validation of the micro-XRF totals on the same samples would be a valuable addition in future work.

The key area of analytical divergence between the two methods—and thus their greatest combined value—lies in elements and signals that one method detects but the other cannot resolve. Three divergences are noteworthy in the present dataset. First, SEM–EDX detected elevated surface-associated C in Biochar + EM (92.7 wt.%) relative to Biochar (77.6 wt.%), a 15.1 wt.% difference that micro-XRF does not measure under the air-acquisition configuration used here, since carbon is below the elemental range routinely quantified by air-coupled micro-XRF (a general technique constraint, not specific to any manufacturer). This surface C signal is consistent with microbial biomass accumulation on biochar surfaces during the 14-day EM incubation^1,19^. Second, SEM–EDX recorded high apparent surface N in the Soil-0 yr spectrum (41.30 wt.%; Table 2), and micro-XRF likewise returned high within-group N variability for the Soil-3 yr and Biochar + EM groups (13.52 ± 23.42 wt.% and 6.69 ± 11.58 wt.% respectively); both observations reflect the unreliability of light-element (N) quantification—by SEM–EDX (semi-quantitative for the N Kα line in standardless quantification) and by micro-XRF in air—rather than a robust nitrogen measurement^26,27^. Third, micro-XRF resolved the transient Fe–Al enrichment at Year 1 across the three field replicates (Fe: 17.77 ± 3.93 wt.%, n = 3 trees), which SEM–EDX corroborated qualitatively for Al (Al = 41.32 wt.% in the Year-1 Map Sum Spectrum; Table 2) but not for Fe (the single-spectrum SEM–EDX Fe rose monotonically through Year 3 rather than peaking at Year 1), reflecting the limits of single-spectrum (n = 1) sampling for resolving temporal trends in spatially heterogeneous Fe phases rather than necessarily indicating divergent geochemistry. These complementary strengths confirm that neither method alone is sufficient to fully characterise the elemental dynamics of biochar-amended soils over multi-year timescales, and that their combined application provides analytical coverage exceeding either method used independently. Independent laboratory analysis (IQS; Supplementary Table S5) gave the bulk total organic carbon (TOC) of the biochar batch as 29.73 wt.% (dry combustion with infrared detection), against which the C-inclusive SEM–EDX Map Sum Spectrum values of 92.7 wt.% (Biochar + EM) and 77.6 wt.% (Biochar) are elevated. Because carbon by standardless EDX on uncoated, carbon-rich biochar is semi-quantitative and prone to over-estimation, and because the surface (EDX) and bulk (combustion) values sample different domains, these surface-to-bulk ratios (~ 3.1 × and ~ 2.6 ×) are indicative rather than absolute. Both biochars show elevated surface carbon, so the higher Biochar + EM value (92.7 vs. 77.6 wt.%) provides qualitative support consistent with greater microbial surface accumulation under EM inoculation^21,22,38^.

The most striking temporal feature was the transient elevation of soil Fe (~ 3.1-fold; 5.68 → 17.77 wt.%) and Al (~ 1.75-fold; 12.19 → 21.33 wt.%) at Year 1, followed by partial decline at Year 2 and secondary increase at Year 3. This non-linear pattern is consistent with dynamic organo-mineral coating formation on freshly applied biochar surfaces, which may serve as nucleation sites for Fe–Al (oxy)hydroxides from soil solution^17,20,38^. The Year 2 decline likely reflects dissolution–reprecipitation equilibria as amorphous phases crystallise, seasonal redox cycling, and competition from accumulating organic matter^39–41^. The secondary Year 3 increase may indicate renewed organo-mineral association driven by seasonal wetting–drying cycles exposing fresh biochar surface area^5,6^. Independent bulk soil total inorganic and organic carbon (TIC/TOC) analysis by combustion elemental analyser (Supplementary Table S8) confirmed a parallel temporal pattern of soil organic carbon (Year 0: 0.83 wt.% → Year 1: 1.57 wt.%; Year 2: 1.25 wt.%; Year 3: 1.87 wt.%), with the transient Year-2 decline and Year-3 recovery directionally aligned with the Fe–Al redistribution dynamics described above.

The Year-1 Fe–Al transient documented here by bulk micro-XRF provides quantitative field-scale evidence for organo-mineral coating dynamics previously visualised only at the nano- to micro-scale. Microspectroscopic studies have shown that biochar surfaces accumulate clay–organic complexes and organo-mineral coatings over time in tropical and subtropical soils, on both outer and inner pore surfaces^21,22,38^. The non-linear Fe–Al dynamics seen here over a shorter 3-year window likely represent an early-stage manifestation of the same process, documented for the first time using routine bulk XRF in a tropical Ultisol. Such coatings also protect the aromatic biochar matrix from oxidative degradation, increasing biochar stability over time^38^–consistent with the partial reversal of Fe–Al at Year 2–3 as coatings stabilise and crystallise.

Because the data are renormalised to a constant sum (carbon- and oxygen-free), the Si fraction varies inversely with the Fe and Al fractions by construction; the net Si decline (77.40 to 50.72 wt.%) is therefore largely the compositional complement of the Year-1 and Year-3 Fe–Al enrichment, with possible secondary contributions from physical dilution by biochar mineral inputs and biochar-mediated adsorption of dissolved silicic acid^18,42^. Ti varied modestly across the chronosequence (1.38–2.04 wt.%) without a statistically significant trend, supporting its use as a relatively conservative lithogenic reference element on the 3-year timescale.

The partial Si recovery at Year 2 supports a mechanism whereby Fe–Al coatings occupy reactive biochar surface sites, temporarily reducing competition for dissolved Si^18,42^.

The absence of measurable micro-XRF differences between Biochar + EM and Biochar (descriptive comparison) is consistent with EM acting through biological rather than mineralogical pathways^1,9,13,43^, consistent with the SEM–EDX evidence for surface C enrichment in Biochar + EM from microbial biofilm deposition during incubation.

Whether this surface-level biological modification influences long-term nutrient release kinetics warrants further investigation. Complementary secondary-electron imaging acquired on the same FESEM at the Maejo IQS facility (Everhart–Thornley detector, 5 kV for soil and 15 kV for biochar, magnification 50–3,000 × ; Sect. “SEM–EDX surface analysis”) supports this interpretation at the structural scale: untreated soil (0Y) showed compact aggregates with abundant bright features consistent with Fe-oxides and limited porosity, whereas biochar-amended soils at 2 and 3 years post-application developed cauliflower-textured macroaggregates (approximately 1–3 mm) with fibrous structures consistent with fungal hyphae and a marked reduction in these bright features. The biochar specimens themselves retained the xylem macropore architecture of the source biomass (approximate lumen diameters 20–60 µm), consistent with biochar acting as a structural template for microbial colonisation and aggregate development. These structural changes, observed independently of the elemental analysis, are mechanistically consistent with the transient Fe–Al maximum at Year 1 followed by partial recovery (the Fe-oxide weathering–coating cycle described above) and with the sustained Ca, K, Mg enrichment at the biochar–soil interface revealed by SEM–EDX.

Principal component analysis of the micro-XRF dataset (Fig. 2) condenses this multi-element response onto two interpretable axes: PC1 (73.1%) separates biochar from soil along a macronutrient (Ca, K, P, Mg)–vs–aluminosilicate (Si, Fe, Al) contrast, and PC2 (12.9%) resolves soil application age in a manner consistent with the Year-1 Fe–Al maximum and subsequent partial recovery described above. The two-component decomposition (86.0% of total variance) provides a compact summary of the elemental dynamics reported in the preceding sections.

Collectively, this multi-element “elemental signature” of biochar aging–resolvable only through combined bulk and surface spectroscopic analysis over multi-year timescales–provides a mechanistic framework for interpreting temporal patterns in soil fertility and nutrient cycling reported in parallel field and incubation studies^1,3,25,43^.

Several limitations warrant acknowledgement. The chronosequence design–sampling different plots at each time point rather than the same plots longitudinally–means between-plot variability cannot be fully excluded; a repeated-measures design would strengthen causal inference. Findings from a single longan-wood biochar in one tropical Ultisol may also not generalise to other feedstocks or soil types, and multi-site replication would improve generalisability. Three analytical constraints further bound interpretation. First, the SEM–EDX dataset is a single Map Sum Spectrum per group (n = 1, six spectra) on different normalisation bases from micro-XRF, so the inter-method comparison is primarily qualitative; the supplementary Spearman concordance reported (Supplementary Table S7) is exploratory and interpreted cautiously given the single SEM–EDX spectrum per group. Second, the Biochar versus Biochar + EM contrast rests on one specimen per treatment in technical triplicate; inferential testing would constitute pseudoreplication, so the absent EM effect on inorganic composition is reported descriptively. Third, light-element values (notably N) are semi-quantitative in both methods and should be read as patterns, not absolute concentrations. Biologically replicated treatments, multi-specimen SEM–EDX on a unified normalisation basis, and independent combustion- or digestion-based N analysis would be needed to test these contrasts inferentially. Beyond the present scope, complementary surface-speciation techniques—notably X-ray photoelectron spectroscopy (XPS) and synchrotron micro-X-ray absorption near-edge structure (µ-XANES)—applied to biochar and biochar–soil mixtures over time would further resolve the surface chemistry and oxidation states of the organo-mineral coatings inferred here.

This three-year field study shows that a single application of nutrient-rich longan-wood biochar drives non-linear, multi-element geochemical redistribution in an acidic tropical Ultisol. The defining result is a transient redistribution of soil Fe and Al that peaked sharply at Year 1, declined at Year 2, and rose again at Year 3—a non-monotonic trajectory that a single time-point measurement would misinterpret as a permanent change, underscoring the need for multiple post-application sampling points in soil elemental monitoring. This Year-1 Fe–Al maximum marks a transient window of greatest phosphate-fixation capacity, when interventions to improve P availability (e.g. liming, additional biochar) are likely to be most effective; the persistent absence of detectable P throughout the chronosequence despite annual fertilisation further confirms that total micro-XRF cannot track plant-available P in Fe/Al-rich tropical soils, for which selective extraction remains necessary. Against this dynamic mineral background, the exceptionally Ca-rich biochar (15 kg tree⁻^1^, ≈ 2.34 t ha⁻^1^) provides a sustained reservoir of Ca, K and Mg and acts as a long-term liming amendment through its alkalinity, although soil Ca and Mg did not change significantly over the three years (only K showed a small measurable trend), consistent with the modest mass added relative to the large soil pool. EM inoculation did not measurably alter the inorganic composition (descriptive comparison), consistent with EM acting through biological rather than mineralogical pathways, while the higher surface carbon detected on Biochar + EM by SEM–EDX points to microbial biofilm accumulation that may shape longer-term nutrient release. Together, the non-linear Fe–Al pulse, inverse Si response, sustained macronutrient enrichment, and the modest K trend form a multi-element “elemental signature” of biochar aging resolvable at field scale—one that combined area-averaged micro-XRF and near-surface SEM–EDX capture more completely than either method alone.

## Methods

### Biochar production and EM inoculation

Two biochar treatments were prepared from longan wood (Dimocarpus longan Lour.) pruning branches as the biomass feedstock. Longan branches were air-dried, cut to uniform size, and subjected to slow pyrolysis at 400–500 °C under limited oxygen conditions. This feedstock was selected due to its local abundance in Chiang Mai, Thailand, where longan orchards are extensively cultivated, and its suitability for producing Ca-rich woody biochar. The resulting biochar was air-dried and stored under ambient conditions prior to treatment preparation.

The effective microorganism (EM) inoculant used for the Biochar + EM treatment was prepared as a secondary EM culture (EM₂) to reduce production cost while maintaining microbial viability. Commercial EM concentrate (EM₁; primary culture) was used as the inoculum source. The EM₂ solution was prepared by first dissolving molasses in dechlorinated water, then adding the EM₁ at a volumetric ratio of EM₁ : molasses : water = 1 : 1 : 18 (v/v/v). The mixture was thoroughly stirred and transferred to sealed plastic containers, leaving approximately 10% headspace to accommodate gas accumulation during fermentation. Containers were incubated at room temperature (25–30 °C) in a shaded area for 7–10 days, with lids briefly opened daily to release fermentation gases. Successful fermentation was confirmed by the presence of a characteristic sweet–sour aroma with no putrid odour, and a dark brown colour with slight turbidity; any batch exhibiting a foul odour was discarded as indicative of contamination. The EM₂ solution was used within three months of preparation to ensure adequate microbial activity.

For the Biochar + EM treatment, the prepared EM₂ solution was diluted to 1 : 200 (v/v) with dechlorinated water and applied to biochar at a rate of 2 L of EM₂ solution per 60 L of biochar (approximately 1 basket unit), either by drenching the biochar mass directly or by soaking the biochar in the diluted EM₂ solution. This application rate was designed to achieve uniform microbial colonisation of biochar pore surfaces. The inoculated biochar was then mixed thoroughly and incubated under covered, ambient conditions for 14 days prior to soil application, allowing establishment of the microbial consortium within the biochar matrix. The second treatment (Biochar) comprised non-inoculated biochar produced from the same batch, processed identically but without EM₂ addition. Both materials were air-dried prior to soil incorporation.

### Soil amendment and sampling design

The study was conducted in longan (Dimocarpus longan Lour.) orchards in Mae Pang Subdistrict, Phrao District, Chiang Mai Province, northern Thailand (~ 19°N, 99°E). Soils belong to the Mae Taeng soil series, classified as Typic Hapludults (Ultisols; USDA Soil Taxonomy), strongly weathered and Fe/Al oxide-rich tropical soils. Biochar was incorporated into the 0–15 cm topsoil layer at a single application rate of 15 kg tree⁻^1^ (≈ 2.34 t ha⁻^1^) at Year 0. Soil samples were collected at four time points: Year 0 (unamended control, S-0 yr), Year 1 (S-1 yr), Year 2 (S-2 yr), and Year 3 (S-3 yr) after biochar incorporation. At each time point, three independent replicate composite samples (n = 3) were collected from the 0–15 cm topsoil layer of separate experimental plots, air-dried at 40 °C, prior to elemental analysis. This is a chronosequence (space-for-time substitution) design: at each post-application time point, three different but agronomically equivalent longan trees were sampled, rather than tracking the same trees longitudinally over three years. All sampled plots were located within the same Mae Taeng soil series, received identical biochar application rate (15 kg tree⁻^1^) at Year 0, and were managed under the same fertiliser regime described above, to minimise between-plot variability inherent to the chronosequence approach. The study comprised six treatment groups with three replicates each (total N = 18 samples). Soil samples were collected from agricultural fields on farmers’ land with their knowledge and consent. No human participants, patient data, or protected or endangered species were involved. The longan (Dimocarpus longan Lour.) pruning branches used for biochar were collected from cultivated orchard trees with the landowners’ permission; longan is a widely cultivated crop rather than a wild or protected species, so no specific collection permits were required, and all plant sampling complied with relevant institutional and national guidelines. Formal institutional ethics committee approval was not required for this field soil survey.

Throughout the study period, all longan orchards received standard fertiliser management according to local farmer practice, as recorded from farmer interviews. Compound chemical fertilisers were applied annually at approximately 300–450 g tree⁻^1^ yr⁻^1^ (300 g at Year 1; ∼340 g at Year 3; 400 g at Year 2; 450 g at Year 0), with organic manure additionally applied in Years 1 and 3. The nutrient totals below are calculated from the compound chemical fertilisers only, as the organic manure composition was not analysed; formulations varied by year: at Year 0 (year of biochar application), a balanced 15–15-15 and 13–13-21 compound fertiliser mix was applied (225 g tree⁻^1^ each; N = 63, P₂O₅ = 63, K₂O = 81 g tree⁻^1^ yr⁻^1^); at Year 1, a lower-rate compound mix (150 g tree⁻^1^ each of 15–15-15 and 13–13-21; N = 42, P₂O₅ = 42, K₂O = 54 g tree⁻^1^ yr⁻^1^) was supplemented with 150 g tree⁻^1^ organic manure; at Year 2, a high-N regime combined 15–15-15 with urea (CO(NH₂)₂; 200 g tree⁻^1^ each; N = 122, P₂O₅ = 30, K₂O = 30 g tree⁻^1^ yr⁻^1^); and at Year 3, a mixed regime of 15–15-15, 13–13-21 and 46–0-0 (∼113 g tree⁻^1^ each; N = 84, P₂O₅ = 32, K₂O = 41 g tree⁻^1^ yr⁻^1^) was applied together with 111 g tree⁻^1^ organic manure. These background nutrient inputs are reported as contextual information; micro-XRF and SEM–EDX elemental analysis was performed on soil and biochar samples and reflects the integrated effect of biochar aging, fertiliser inputs, and natural soil processes over the study period.

### micro-XRF elemental analysis

Multi-element composition was determined by energy-dispersive micro-X-ray fluorescence (micro-XRF), a spatially-resolved technique using a benchtop spectrometer fitted with a rhodium (Rh) anode microfocus X-ray tube and polycapillary focusing optics. Each air-dried sample was analysed directly as an intact specimen, without grinding or pelletisation, with the specimen surface presented to the measurement spot^14,15,27^. Measurements were performed under ambient air (no vacuum or helium purge) at a tube voltage of 50 kV and a tube current of 600 µA. For each specimen a two-dimensional elemental map was acquired over a field of view of approximately 2–5 mm at a pixel spacing (step size) of 20 µm and a dwell time of 20–25 ms per pixel; the total acquisition time per map (~ 15 min) was computed automatically from the mapped area and step size. Elemental concentrations were derived from the area (Map Sum) spectrum and quantified using the standardless fundamental-parameters routine of the instrument software. Results are reported as normalised weight percent over the detected elements; carbon and oxygen are not quantified in this configuration, so values are expressed on a carbon- and oxygen-free basis. Because measurements were acquired in air, the low-energy lines of light elements (notably N, and to a lesser degree Na, Mg, Al, Si, P and S) are strongly attenuated and their values are semi-quantitative.

The replication structure differed between materials. For each soil application age (0, 1, 2 and 3 years), three independent field replicates were analysed, one from each of three longan trees (topsoil 0–15 cm), giving twelve independent soil samples; the three values per age are therefore independent field replicates. For each biochar treatment (Biochar and Biochar + EM), a single representative specimen was measured three times; these are technical replicates reflecting measurement repeatability. Values are reported as mean ± standard deviation, and this distinction is carried through to the statistical analysis.

### SEM–EDX surface analysis

Surface morphology and localised elemental composition were characterised by field-emission scanning electron microscopy (FESEM) coupled with energy-dispersive X-ray spectroscopy (EDX) using a silicon drift detector and vendor-supplied software with standardless ZAF correction. A focused electron beam generates characteristic X-rays from the sample, which are collected by the silicon drift detector and quantified by standardless matrix correction. EDX analysis was performed at 15 kV, a working distance of approximately 10 mm, and a beam current of approximately 300 pA–1 nA. For each of the six sample groups (Biochar, Biochar + EM, and Soil at 0, 1, 2 and 3 years post-application), a single Map Sum Spectrum was acquired from a representative field of view approximately 0.5–3 mm across, at low-to-moderate magnification, yielding six EDX spectra in total (n = 1 per group). The two biochar Map Sum Spectra were retained on the carbon-inclusive basis to demonstrate surface organic composition (Biochar + EM = 92.7 wt.% C; Biochar = 77.6 wt.% C); the four soil Map Sum Spectra are reported on the carbon- and oxygen-free renormalisation basis to emphasise the inorganic mineral fraction. Quantitative inter-group variability and statistical inference were addressed by the micro-XRF analysis (Table 1, n = 3 per group); the SEM–EDX Map Sum Spectra are therefore presented as single-spectrum qualitative demonstrations of complementary near-surface composition rather than as a basis for inferential statistics. At 15 kV the electron interaction volume is shallow (~ 1–2 µm), so EDX reports micrometre-scale near-surface composition, in contrast to the millimetre-scale area averaging of micro-XRF. Because the SEM–EDX dataset comprises a single spectrum per group, no within-group dispersion or significance testing is reported for SEM–EDX; instead, the role of SEM–EDX in this study is to provide a spatially complementary view of the elemental composition resolved by micro-XRF. The light-element EDX channels (N, O) are treated qualitatively, since quantification of these channels by any standardless EDX system on a renormalised basis is inherently sensitive to peak-overlap and background-fitting conventions; this is a well-known characteristic of the standardless quantification methodology and is not specific to any particular EDX manufacturer or detector.

Soil samples were mounted on aluminium stubs with conductive carbon adhesive tape and sputter-coated with an approximately 5 nm gold (Au) layer in a high-vacuum sputter coater to minimise surface charging; biochar samples were mounted on aluminium stubs without conductive coating, as the pyrogenic carbon matrix is naturally conductive. Unlike micro-XRF, SEM–EDX directly quantifies carbon and oxygen; all SEM–EDX values are normalised to 100 wt.% over the detected elements, on the bases described above. Independent verification of the bulk biochar composition was obtained from the Institute of Product Quality and Standardization (IQS), Maejo University, Thailand, an accredited testing facility. A single representative sample of the longan-wood biochar batch used for the field application was analysed for total nitrogen by the Kjeldahl method (Department of Agriculture Manual for Organic Fertiliser Analysis, Thailand, 2008); total carbon and total inorganic carbon by dry combustion with infrared detection (DIN 51,732, ISO 29,541, DIN 51,726, ISO 925); phosphorus (as P₂O₅), potassium (as K₂O), and total Ca and Mg by inductively-coupled plasma optical-emission spectrometry (ICP-OES) following microwave-assisted acid digestion (US EPA method 3051A); and ash content by ASTM D1762-84. The IQS reference values are reported in Supplementary Table S5 and used as bulk-composition reference points for the micro-XRF and SEM–EDX measurements.

In addition to EDX acquisition, the same FESEM at the Maejo IQS facility was operated in secondary-electron (SE) imaging mode using the built-in Everhart–Thornley detector to document micromorphology of all six sample groups (n = 1 per group). Soil specimens were imaged at an accelerating voltage of 5 kV and biochar specimens at 15 kV, with working distances of 9.0–11.0 mm and magnifications spanning 50 × (field of view 7.45 mm) to 3,000 × (field of view 124 µm), covering aggregate-scale structure (> 1 mm) to grain-scale microporosity (< 5 µm). Probe currents were approximately 40–60 pA with pixel dwell times of 3.2 µs for soil and up to 32 µs for biochar; all imaging was performed in high-vacuum mode (chamber pressure 0.6–12.3 mPa) using the instrument’s vendor-supplied imaging software (acquisition sessions: 28 November and 2 December 2025). SE images are reported as qualitative micromorphological observations only and are not used for quantitative inference; the EDX dataset above provides the elemental information.

### Statistical analysis

Descriptive statistics (mean ± s.d.) were calculated for all micro-XRF and SEM–EDX measurements. The choice of inferential test followed the replication structure (Sect. “Soil amendment and sampling design”). For the soil series, the three replicates per application age are independent field replicates (three trees), so differences in elemental composition among the four application ages (0, 1, 2 and 3 years) were tested by one-way analysis of variance (ANOVA; α = 0.05) after checking normality with the Shapiro–Wilk test (Supplementary Table S3); this applies to the principal temporal findings, including the transient Year-1 Fe–Al maximum and the Si and K trends. The Biochar versus Biochar + EM comparison is based on a single specimen per treatment measured three times (technical replicates); inferential testing of this contrast would constitute pseudoreplication, so it is reported descriptively only (mean ± s.d.), without p-values. Light-element values (notably N) are excluded from inferential analysis given their semi-quantitative nature in air (Sect. “micro-XRF elemental analysis”). The micro-XRF and SEM–EDX datasets are compared primarily qualitatively across spatial scales, because the SEM–EDX data comprise a single unreplicated spectrum per group (biochars carbon-inclusive; soils renormalised carbon- and oxygen-free) whereas micro-XRF provides area-averaged values on a carbon- and oxygen-free basis; a supplementary Spearman rank correlation between the two datasets (Supplementary Table S7) is reported as an exploratory concordance check, interpreted cautiously given the single SEM–EDX spectrum per group. Principal component analysis (PCA) was performed on the micro-XRF elemental composition data using the scikit-learn v1.3 implementation of singular value decomposition (SVD), with input data standardised to zero mean and unit variance (StandardScaler) prior to decomposition; PC1 (73.1%) and PC2 (12.9%) together explained 86.0% of the total variance and are reported here (full loadings in Supplementary Table S4). Given the modest sample size (n = 18), unsupervised PCA was preferred over supervised machine-learning classifiers, which would be prone to over-fitting at this number of observations; PCA provides a robust, assumption-light summary of the multi-element covariance structure appropriate to a dataset of this size. All analyses were conducted in Python 3.11 (NumPy v1.24, SciPy v1.10, scikit-learn v1.3, pandas v2.0, matplotlib v3.7).

### AI tool use

Artificial intelligence (AI) tools were used to help with language editing and rephrasing while preparing this manuscript. The data, analysis, interpretation, and conclusions are the authors’ own work, for which the authors take full responsibility. This use is disclosed in line with the policy of Scientific Reports.

## Supplementary Information

Below is the link to the electronic supplementary material.

Supplementary Material 1.

Supplementary Material 2.

Supplementary Material 3.

## Data Availability

“Data available from the corresponding author on request”.
