# Supplementary material for: Temporal dynamics of nutrient elements in biochar and biochar-amended soils over three years: a comparative micro-XRF and SEM–EDX study
Source: Sci Rep. 2026 Jun 21;16:22048. doi: 10.1038/s41598-026-59314-z (PMC13370010; doi:10.1038/s41598-026-59314-z)
Supplement: Supplementary file 1 — Supplementary Material 1. [file 41598_2026_59314_MOESM1_ESM.pdf]

Supplementary Information

Temporal Dynamics of Nutrient Elements in Biochar and Biochar-Amended Soils over Three Years:

A Comparative micro-XRF and SEM-EDX Study

Suphathida Aumtong, Phruetthiphong Soongsoongnoen, Dechatorn Wanwinit

This file contains eight supplementary tables and one supplementary figure:

Table S1 — Complete micro-XRF elemental composition (6 treatment groups × 11 elements, mean ± s.d.)

Table S2 — micro-XRF vs SEM-EDX comparison (all 6 groups × 8 shared elements, on the carbon- and oxygen-free normalisation basis)

Table S3 — Shapiro–Wilk normality test results for all micro-XRF measurements

Table S4 — Principal component analysis (PCA) loadings for PC1 and PC2

Table S5 — Independent IQS laboratory analysis of the biochar batch (Kjeldahl, ICP-OES, dry combustion, ASTM D1762)

Table S6 — Extractable plant-available P (mg kg^{-1}) in topsoil (0–15 cm) by period of biochar use. Values are mean ± standard deviation (n = 9 field samples per period). The increase by Year 3 was not statistically significant (one-way ANOVA, $F(3,32) = 1.82$, $p = 0.16$, $\eta^2 = 0.15$). These extractable-P data complement the micro-XRF total-P results (below detection), illustrating the distinction between total and plant-available P.

Table S7 — Paired micro-XRF and SEM-EDX values (n = 48) underlying the Spearman rank-concordance analysis

Table S8 — Bulk soil total organic carbon (TOC, wt.%) by period of biochar use (dry-combustion elemental analysis).

Figure S1 — Macronutrient concentrations (Ca, K, P, Mg) of all six treatment groups by micro-XRF

Supplementary Table S1. Complete micro-XRF elemental composition of all treatment groups.

All values are wt.% (mean ± s.d., n = 3 per group). Values at or below the micro-XRF detection limit are reported as 0.00. The Biochar and Biochar+EM measurements are technical replicates of a single specimen; the four soil groups are field replicates from three trees per soil age.

**Biochar +EM**	62.16 ± 6.19	11.76 ± 6.93	5.29 ± 1.80	6.30 ± 1.01	5.32 ± 1.77	0.99 ± 0.66	0.51 ± 0.12	6.69 ± 11.58	0.18 ± 0.11	0.12 ± 0.01	0.48 ± 0.24
**Biochar**	60.39 ± 4.61	15.85 ± 4.27	5.62 ± 0.37	6.66 ± 0.36	5.72 ± 0.80	0.88 ± 0.42	0.55 ± 0.07	3.45 ± 5.98	0.20 ± 0.04	0.10 ± 0.01	0.31 ± 0.03
**Soil 0yr**	0.61 ± 0.57	1.48 ± 0.07	0.00	0.15 ± 0.03	77.40 ± 0.70	5.68 ± 1.02	12.19 ± 0.52	0.00	2.04 ± 0.11	0.25 ± 0.05	0.00
**Soil 1yr**	0.85 ± 0.06	1.57 ± 0.02	0.00	0.16 ± 0.01	55.77 ± 5.39	17.77 ± 3.93	21.33 ± 1.88	0.00	1.95 ± 0.29	0.20 ± 0.17	0.00
**Soil 2yr**	0.63 ± 0.17	1.82 ± 0.07	0.00	0.12 ± 0.10	73.12 ± 7.31	8.30 ± 4.09	13.34 ± 3.22	0.00	1.99 ± 0.15	0.30 ± 0.07	0.00
**Soil 3yr**	0.56 ± 0	1.49 ± 0	0.00	0.14 ± 0	50.72 ±	13.74 ±	18.09 ±	13.52 ±	1.38 ± 0	0.10 ± 0	0.01 ± 0

	.48	.21		.05	15.18	4.60	3.43	23.42	.44	.12	.01

Supplementary Table S2. Direct side-by-side comparison of micro-XRF (Table 1) and SEM-EDX values for the same treatment groups, on the carbon- and oxygen-free renormalisation basis.

micro-XRF: bulk-area scan of intact specimens at ambient pressure (energy-dispersive micro-XRF; mean ± s.d. of n = 3 replicate spectra per group, on the C- and O-free renormalisation basis). SEM-EDX: single Map Sum Spectrum per group (FESEM with silicon drift EDX detector and standardless quantification; n = 1 per group, area-averaged across a representative field of view approximately 0.5–3 mm across at low-to-moderate magnification, on the C- and O-free renormalisation basis). Because SEM-EDX is single-spectrum per group, no within-group dispersion is reported and no inferential statistics are performed on SEM-EDX values; Table S2 provides a direct visual comparison only. n.d. = element not detected in the SEM-EDX spectrum.

**Biochar+EM**	micro-XRF	62.16 ± 6.19	11.76 ± 6.93	5.29 ± 1.80	6.30 ± 1.01	5.32 ± 1.77	0.99 ± 0.66	0.51 ± 0.12	6.69 ± 11.58
	SEM-EDX	43.84	12.33	15.07	15.07	12.33	n.d.	n.d.	n.d.
**Biochar**	micro-XRF	60.39 ± 4.61	15.85 ± 4.27	5.62 ± 0.37	6.66 ± 0.36	5.72 ± 0.80	0.88 ± 0.42	0.55 ± 0.07	3.45 ± 5.98
	SEM-EDX	33.33	20.29	18.84	14.49	11.59	n.d.	1.45	n.d.
**Soil 0yr**	micro-XRF	0.61 ± 0.57	1.48 ± 0.07	0.00	0.15 ± 0.03	77.40 ± 0.70	5.68 ± 1.02	12.19 ± 0.52	0.00
	SEM-EDX	0.30	0.70	0.00	0.70	34.10	2.90	17.60	41.30
**Soil 1yr**	micro-XRF	0.85 ± 0.06	1.57 ± 0.02	0.00	0.16 ± 0.01	55.77 ± 5.39	17.77 ± 3.93	21.33 ± 1.88	0.00
	SEM-EDX	0.49	n.d.	0.00	1.22	51.34	6.11	41.32	n.d.
**Soil 2yr**	micro-XRF	0.63 ± 0.17	1.82 ± 0.07	0.00	0.12 ± 0.10	73.12 ± 7.31	8.30 ± 4.09	13.34 ± 3.22	0.00
	SEM-EDX	0.60	n.d.	0.00	2.30	86.50	7.60	n.d.	n.d.
**Soil 3yr**	micro-XRF	0.56 ± 0.48	1.49 ± 0.21	0.00	0.14 ± 0.05	50.72 ± 15.18	13.74 ± 4.60	18.09 ± 3.43	13.52 ± 23.42
	SEM-EDX	1.10	n.d.	0.00	3.40	78.10	17.00	n.d.	0.00

Supplementary Table S3. Shapiro–Wilk normality test results for micro-XRF measurements.

W statistic and p-value computed on the three replicate measurements per treatment group for each element. Elements with all-zero values (below detection limit in all three replicates) are omitted. Entries marked * indicate departure from normality at α = 0.05; these involve elements with one large outlier replicate and two zero or near-zero replicates (typical of light-element semi-quantitative artefacts).

**Biochar+EM**	Ca	0.791	0.0942	Yes
**Biochar+EM**	K	0.804	0.1241	Yes
**Biochar+EM**	P	0.764	0.0318	No *
**Biochar+EM**	Mg	0.775	0.0570	Yes
**Biochar+EM**	Si	0.757	0.0162	No *
**Biochar+EM**	Fe	0.776	0.0580	Yes
**Biochar+EM**	Al	0.750	< 0.001	No *
**Biochar+EM**	N	0.750	< 0.001	No *
**Biochar+EM**	Ti	0.828	0.1832	Yes
**Biochar+EM**	Mn	0.750	< 0.001	No *
**Biochar+EM**	S	0.786	0.0806	Yes
**Biochar**	Ca	0.999	0.9461	Yes

**Biochar**	K	0.897	0.3758	Yes
**Biochar**	P	0.917	0.4418	Yes
**Biochar**	Mg	0.887	0.3458	Yes
**Biochar**	Si	0.973	0.6868	Yes
**Biochar**	Fe	0.978	0.7134	Yes
**Biochar**	Al	0.812	0.1436	Yes
**Biochar**	N	0.750	< 0.001	No *
**Biochar**	Ti	1.000	1.0000	Yes
**Biochar**	Mn	1.000	1.0000	Yes
**Biochar**	S	0.750	< 0.001	No *
**Soil 0yr**	Ca	0.758	0.0168	No *
**Soil 0yr**	K	0.812	0.1436	Yes
**Soil 0yr**	Mg	0.987	0.7804	Yes
**Soil 0yr**	Si	0.935	0.5085	Yes
**Soil 0yr**	Fe	0.783	0.0747	Yes
**Soil 0yr**	Al	0.856	0.2557	Yes
**Soil 0yr**	Ti	0.860	0.2665	Yes
**Soil 0yr**	Mn	0.750	< 0.001	No *
**Soil 1yr**	Ca	0.824	0.1736	Yes
**Soil 1yr**	K	0.964	0.6369	Yes
**Soil 1yr**	Mg	1.000	1.0000	Yes
**Soil 1yr**	Si	0.774	0.0531	Yes
**Soil 1yr**	Fe	0.848	0.2341	Yes
**Soil 1yr**	Al	0.832	0.1933	Yes
**Soil 1yr**	Ti	0.750	< 0.001	No *
**Soil 1yr**	Mn	0.864	0.2783	Yes
**Soil 2yr**	Ca	0.982	0.7421	Yes
**Soil 2yr**	K	0.983	0.7470	Yes
**Soil 2yr**	Mg	0.865	0.2815	Yes
**Soil 2yr**	Si	0.777	0.0601	Yes
**Soil 2yr**	Fe	0.772	0.0490	No *
**Soil 2yr**	Al	0.831	0.1901	Yes
**Soil 2yr**	Ti	0.858	0.2630	Yes
**Soil 2yr**	Mn	0.953	0.5827	Yes
**Soil 3yr**	Ca	0.893	0.3631	Yes
**Soil 3yr**	K	0.974	0.6878	Yes
**Soil 3yr**	Mg	0.987	0.7804	Yes
**Soil 3yr**	Si	0.881	0.3275	Yes
**Soil 3yr**	Fe	0.994	0.8561	Yes
**Soil 3yr**	Al	0.970	0.6674	Yes
**Soil 3yr**	N	0.750	< 0.001	No *
**Soil 3yr**	Ti	0.788	0.0870	Yes
**Soil 3yr**	Mn	0.970	0.6678	Yes
**Soil 3yr**	S	0.750	< 0.001	No *

Supplementary Table S4. PCA loadings for PC1 and PC2.

PCA was performed on the standardised composition (zero mean, unit variance per element) of all 11 detected elements (Ca, K, P, Mg, Si, Fe, Al, N, Ti, Mn, S) across the 18 samples, using the scikit-learn singular-value-decomposition implementation; loadings are the standardised eigenvector coefficients of each element on PC1 and PC2. Loadings with |value| ≥ 0.25 (highlighted in the biplot, Fig. 2b) are considered substantial. PC1 + PC2 together explain 86.0 % of the total variance (PC1: 73.1 %; PC2: 12.9 %), in agreement with the scree plot in Fig. 2a. PC1 separates the Ca/P/Mg/K/S-rich biochar materials (positive) from the Si/Ti/Al/Fe-rich soil matrix (negative); PC2 is dominated by nitrogen, with manganese loading in the opposite sense, capturing the two samples with anomalous semi-quantitative N (Biochar+EM rep 1 and Soil 3yr rep 1).

**Ca**	+0.35	+0.06	Strong positive PC1 (biochar Ca-enrichment axis)
**P**	+0.35	+0.09	Positive PC1 (biochar nutrient signal)
**Mg**	+0.34	+0.01	Positive PC1 (biochar nutrient signal)
**K**	+0.32	+0.14	Positive PC1 (biochar nutrient signal)
**S**	+0.33	+0.10	Positive PC1 (biochar nutrient signal)
**N**	+0.05	−0.62	Near-zero PC1; dominant negative PC2 (semi-quantitative N axis)
**Mn**	−0.17	+0.59	Negative PC1; strong positive PC2
**Fe**	−0.27	−0.31	Negative PC1 (soil Fe-oxide matrix)
**Al**	−0.32	−0.19	Strong negative PC1 (soil silicate/oxide matrix)
**Si**	−0.33	+0.23	Strong negative PC1 (soil silicate matrix)
**Ti**	−0.34	+0.20	Strong negative PC1 (soil mineral)
**Variance explained**	73.1 %	12.9 %	PC1 + PC2 = 86.0 % cumulative

Supplementary Table S5. Independent IQS laboratory analysis of the bulk biochar batch used in the field application.

A single representative sample of the longan-wood biochar batch (the same material applied to all soil plots) was analysed by the Institute of Product Quality and Standardization (IQS), Maejo University, Thailand, an accredited testing facility. Values serve as independent bulk-composition reference points against which the non-destructive micro-XRF and SEM-EDX measurements in the main text can be compared. Methods follow national and international standards as indicated below.

**Total nitrogen (N)**	0.79	wt. %	Kjeldahl digestion (Department of Agriculture Manual for Organic Fertiliser Analysis, Thailand, 2008)
**Phosphorus (as P_2O_5)**	0.68	wt. %	ICP-OES after microwave-assisted acid digestion (US EPA method 3051A; in-house method T-011)
**Potassium (as K_2O)**	1.27	wt. %	ICP-OES after microwave-assisted acid digestion (US EPA method 3051A; in-house method T-011)
**Total carbon (TC)**	29.84	wt. %	Dry combustion with infrared detection (DIN 51732, ISO 29541, DIN 51726, ISO 925)
**Total inorganic carbon (TIC)**	0.11	wt. %	Dry combustion with infrared detection (same as TC)
**Total organic carbon (TOC)**	29.73	wt. %	Calculated: TOC = TC − TIC
**Calcium (total Ca)**	3.79 (37,882 mg/kg)	wt. %	ICP-OES after microwave-assisted acid digestion (US EPA method 3051A; in-house

			method T-011)
**Magnesium (total Mg)**	0.39 (3,879 mg/kg)	wt. %	ICP-OES after microwave-assisted acid digestion (US EPA method 3051A; in-house method T-011)
**Ash content**	8.73	wt. %	ASTM D1762-84 (Standard Test Method for Chemical Analysis of Wood Charcoal)

Notes on Table S5: The IQS analysis was performed on a single homogenised representative sample of the biochar batch; no replication was performed at the IQS laboratory. The values are reported here to provide independent bulk-composition reference points and are compared against the multi-replicate micro-XRF and SEM-EDX values in the main text (Methods §4.4; Discussion). The relationship Kjeldahl-N (0.79 wt.%) ≪ micro-XRF-N in air (3.45–13.52 wt.%), together with the implausibly high SEM-EDX N recorded in one soil spectrum (41.30 wt.%, Soil 0yr), confirms that the spectroscopic N values are semi-quantitative artefacts rather than absolute concentrations (see Newbury & Ritchie 2015, ref. [26]; Fedeli et al. 2024, ref. [27]). The relationship bulk TOC (29.73 wt.%) ≪ SEM-EDX surface C on the C-inclusive basis (77.6–92.7 wt.%) confirms substantial surface-localised carbon enrichment on biochar particles consistent with the formation of microbial biofilm and/or extracellular polymeric substances on Biochar+EM after the 14-day EM incubation (Hagemann et al. 2017, ref. [38]; Joseph et al. 2022, ref. [21]; Lin et al. 2012, ref. [22]).

Supplementary Table S6. Extractable plant-available P (mg kg^{-1}) in topsoil (0–15 cm) by period of biochar use. Values are mean ± standard deviation (n = 9 field samples per period). The increase by Year 3 was not statistically significant (one-way ANOVA, $F(3,32) = 1.82$, $p = 0.16$, $\eta^2 = 0.15$). These extractable-P data complement the micro-XRF total-P results (below detection), illustrating the distinction between total and plant-available P.

Period of biochar use	Available P (mg kg^{-1}), mean ± SD	n
**Year 0**	210 ± 156	9
**Year 1**	261 ± 288	9
**Year 2**	211 ± 163	9
**Year 3**	466 ± 398	9

Supplementary Table S7. Paired micro-XRF and SEM-EDX elemental values (n = 48; 8 overlapping elements across 6 sample groups) underlying the Spearman rank-concordance analysis.

All values are on the carbon- and oxygen-free renormalisation basis (wt.%). micro-XRF values are the mean ± s.d. of n = 3 replicate spectra per group (Table S1); the Spearman analysis was computed on these group means. SEM-EDX values are single Map Sum Spectra (n = 1 per group; no within-group dispersion). “n.d.” = element not detected in the SEM-EDX spectrum; “0.00” = within the analytical window but below quantifiable signal. A “dual-detected pair” is one in which both methods recorded a value above zero for that group; the 30 dual-detected pairs constitute the restricted subset.

**Biochar+EM**	Ca	62.16 ± 6.19	43.84	Yes
	K	11.76 ± 6.93	12.33	Yes
	P	5.29 ± 1.80	15.07	Yes
	Mg	6.30 ± 1.01	15.07	Yes
	Si	5.32 ± 1.77	12.33	Yes
	Fe	0.99 ± 0.66	*n.d.*	—
	Al	0.51 ± 0.12	*n.d.*	—
	N	6.69 ± 11.58	*n.d.*	—
**Biochar**	Ca	60.39 ± 4.61	33.33	Yes
	K	15.85 ± 4.27	20.29	Yes
	P	5.62 ± 0.37	18.84	Yes

	Mg	6.66 ± 0.36	14.49	Yes
	Si	5.72 ± 0.80	11.59	Yes
	Fe	0.88 ± 0.42	*n.d.*	—
	Al	0.55 ± 0.07	1.45	Yes
	N	3.45 ± 5.98	*n.d.*	—
**Soil 0yr**	Ca	0.61 ± 0.57	0.30	Yes
	K	1.48 ± 0.07	0.70	Yes
	P	0.00	0.00	—
	Mg	0.15 ± 0.03	0.70	Yes
	Si	77.40 ± 0.70	34.10	Yes
	Fe	5.68 ± 1.02	2.90	Yes
	Al	12.19 ± 0.52	17.60	Yes
	N	0.00	41.30	—
**Soil 1yr**	Ca	0.85 ± 0.06	0.49	Yes
	K	1.57 ± 0.02	*n.d.*	—
	P	0.00	0.00	—
	Mg	0.16 ± 0.01	1.22	Yes
	Si	55.77 ± 5.39	51.34	Yes
	Fe	17.77 ± 3.93	6.11	Yes
	Al	21.33 ± 1.88	41.32	Yes
	N	0.00	*n.d.*	—
**Soil 2yr**	Ca	0.63 ± 0.17	0.60	Yes
	K	1.82 ± 0.07	*n.d.*	—
	P	0.00	0.00	—
	Mg	0.12 ± 0.10	2.30	Yes
	Si	73.12 ± 7.31	86.50	Yes
	Fe	8.30 ± 4.09	7.60	Yes
	Al	13.34 ± 3.22	*n.d.*	—
	N	0.00	*n.d.*	—
**Soil 3yr**	Ca	0.56 ± 0.48	1.10	Yes
	K	1.49 ± 0.21	*n.d.*	—
	P	0.00	0.00	—
	Mg	0.14 ± 0.05	3.40	Yes
	Si	50.72 ± 15.18	78.10	Yes
	Fe	13.74 ± 4.60	17.00	Yes
	Al	18.09 ± 3.43	*n.d.*	—
	N	13.52 ± 23.42	0.00	—

Spearman rank-concordance results. Across all 48 pairs (n.d. coded as zero): $\rho = 0.585$, $p < 1 \times 10^{-4}$. Restricted to the 30 dual-detected pairs: $\rho = 0.844$, $p < 1 \times 10^{-8}$. For reference, the parametric Pearson correlation across the same 48 pairs was $r = 0.809$ ($p < 1 \times 10^{-11}$). The moderate-to-strong, element-dependent concordance indicates that bulk micro-XRF and near-surface SEM-EDX provide complementary rather than interchangeable compositional information; a near-unity inter-method correlation (e.g. $r \approx 0.97$) is not supported by this dataset.

Supplementary Table S8. Bulk soil total organic carbon (TOC, wt.%) by period of biochar use, determined independently by dry-combustion elemental analysis (separate from the micro-XRF dataset). The non-monotonic pattern (Year-1 rise, Year-2 decline, Year-3 recovery) is directionally aligned with the micro-XRF Fe–Al redistribution dynamics described in the main text. Inorganic carbon (TIC) was negligible in these acidic soils.

Period of biochar use	Soil organic carbon (TOC, wt.%)
**Year 0**	0.83

**Year 1**	1.57
**Year 2**	1.25
**Year 3**	1.87

Supplementary Figure S1. Macronutrient concentrations of all treatment groups by micro-XRF.

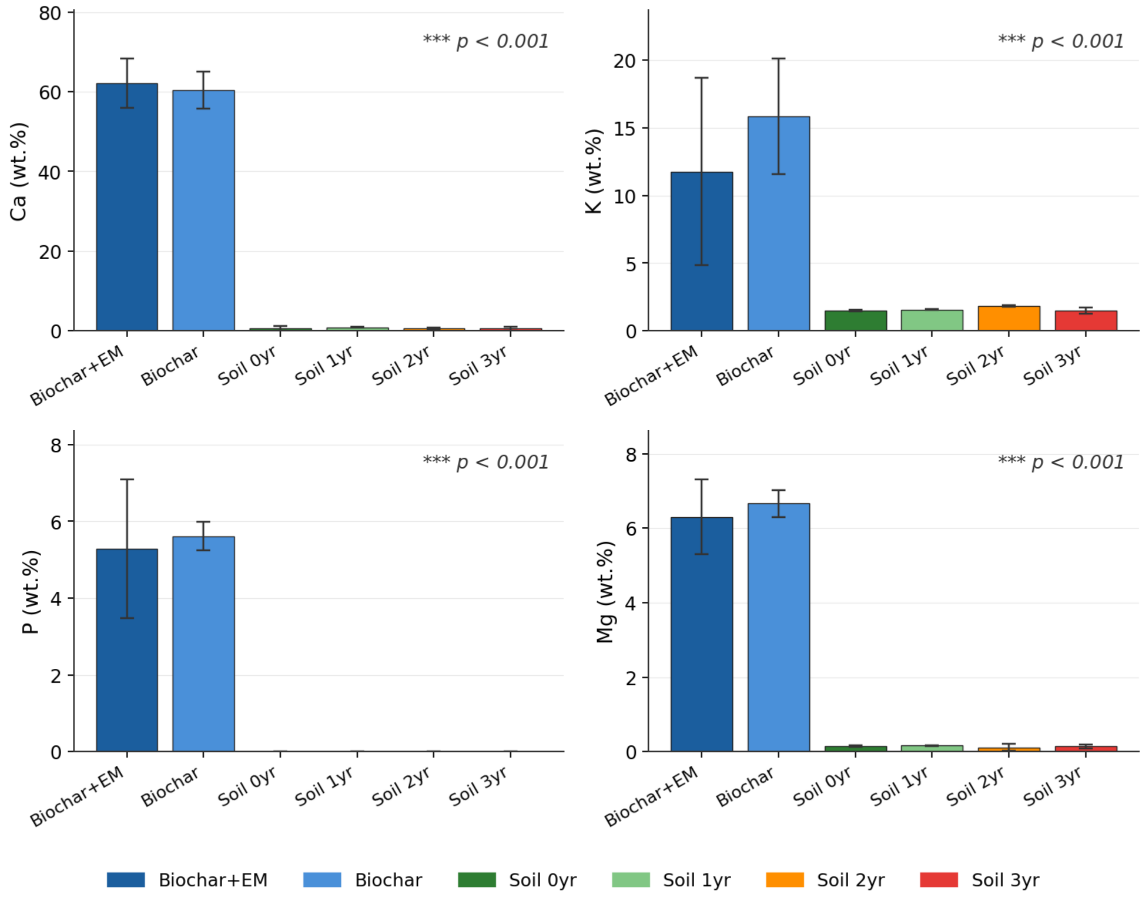

Bar charts of Ca, K, P and Mg (normalised wt.%) for all six treatment groups; error bars = s.d. (n = 3). Soil-only one-way ANOVA (df = 3, 8) showed a significant difference among soil application ages only for K ($p = 0.024$); Ca and Mg did not differ significantly, and P was below the micro-XRF detection limit in all soil groups. The biochar-versus-soil contrast is descriptive only (single specimen per biochar). Values correspond to the micro-XRF data in Supplementary Table S1 and main-text Table 1.
